# Address to the Graduating Class of the Ohio Dental College

**Published:** 1867-03

**Authors:** Jos. Richardson


### DENTAL REGISTER.

VOL. XXI.] MARCH, 1867. [No. 3

### Original Communications.

### ADDRESS TO THE GRADUATING CLASS OF THE OHIO DENTAL COLLEGE, May 6th, 1867.

BY JOS. RICHARDSON, M. D., D. D. S.

*Gentlemen of the Graduating Class:*—The friendly partiality of the faculty has assigned me the agreeable duty of addressing to you this evening, a few words of congratulation on the completion of your collegiate course, and of salutation on your entrance into the profession, clothed as you now are with all the rights, dignities, and immunities conferred by the degree of Doctor of Dental Surgery. I esteem it a privilege of no ordinary character, that of greeting you upon the very threshold, and saying to you, in behalf of the faculty, and of the profession: Welcome to the brotherhood of Dentistry.

In conforming to this customary and somewhat formal mode of extending to you assurances of good-will and hearty fellowship, we desire especially, that you will accept our fraternal protestations, not in the sense of mere conventional civilities, but as expressions of unstudied, earnest and warm-hearted greeting. The occasion which ushers into the profession, men fully impressed with a just sense of its grave responsibilities, and adequately qualified to discharge creditably its varied and important duties, has always been esteemed one of peculiar interest; but never more than now, perhaps, has been invested with greater significances, or more gratefully accepted by all good men with sentiments of more profound approbation and satisfaction.

While we are not insensible of the fact, that material and encouraging progress in all that relates to the resources and capabilities of the art and science of Dentistry has been made, and that intelligent, earnest and wisely-directed efforts have steadily contributed to render our calling more and more a blessing to mankind; yet it may not be unprofitable at this time, to familiarize ourselves with the indisputable fact, that at no previous time has the profession suffered more cruel wrongs at the hands of adventurers and incompetents, than now. You will all soon realize this unpalatable truth, in the respective localities which you may severally choose, be they where they may throughout this broad land. So fruitful is the field of Dental practice in opportunities for downright swindling, and so comparatively secure from public reprobation, and legal accountability are the miscreants who seek this field for purely mercenary ends, that quacks will always flourish, and fatten, and preponderate, at least until a higher order of intelligence and discernment prevails among the masses, than is likely to in this nineteenth century, or until the majesty of the law is invoked to shield the people, and guard the temple of our art and science, from desecration and outrage.

Contemplating the magnitude of the evils inflicted upon the highest interests of the profession, and the unspeakable wrongs entailed upon the people, by miserable wretches, who having no conceivable morals, mental or educational qualifications for the practice, are hourly prostituting Dentistry to the most unworthy and dishonorable purposes, is it at all singular, that those who are jealous of the fair name of the profession, and who are striving with more than ordinary singleness of purpose to make it honored and respected among men, should regard with exultation, occasions like this, which yearly contribute to that profession new life and hope, or that they should greet with unaffected pride and satisfaction, these annual accessions to the ranks of the faithful.

It was a just and conscientious estimate of duty, as men answerable to God and society, that led you to seek the advantages of this institution. It was an honorable ambition, alike creditable to your heart and manhood, that directed your steps to this sanctuary of learning, that you might fortify yourselves for the better discharge of your assumed obligations in the coming time. It was a laudable and worthy motive, that prompted the dedication of time and means, that you might hereafter win your way to honorable distinction among your fellows, by laying the foundation of success here.

We should be most happy, gentlemen, if we could impart to you the comforting assurance, that your just claims to the confidence and support of the people, will meet with a ready and unquestioned recognition, but we cannot, knowing that you will have to fight your way inch by inch with those who are not only disqualified by nature and education for the practice, but who are unscrupulous in the choice of means to render their pretensions successful. It is indeed a sorrowful and humiliating reflection, that you, who have been so carefully nurtured here in this school, and who have so worthily responded to the uttermost demands of the profession, should be exposed hereafter to the unworthy competition of upstart Dentists, a score of whom will confront you at every point, with their spurious claims to public confidence and patronage. It is a sense of this great and palpable wrong, that has of late moved the profession, almost as one man, to seek protection in prohibitory legislation. Believing that you will all recognize the obligation to contribute the weight of your influence in behalf of some measure contemplating this object, we trust you will indulge us if we devote a portion of our time this evening to a brief consideration of this subject.

Bills, differing somewhat in detail, but embracing the same general features, have been submitted to the legislatures of several of the States. Those of Ohio and Indiana prescribe that no one shall be deemed competent to practice Dentistry, except those who are graduates of some regularly incorporated Dental college; those who have been ten years in reputable practice; or those holding a certificate of qualification, from a board of examiners, appointed by the Governor of the State.

It would seem almost a work of supererogation, to advocate the claims of such a law before an assemblage of Dentists, and it is only in the hope that we may be mutually strengthened in its advocacy before the legislative bodies, that we are tempted to enter upon its discussion at this time, trusting we may be able to present some thoughts that may hereafter aid you in prosecuting the objects contemplated in the proposed law.

That certain misapprehensions, fatal to the success of such a measure, exist in the minds of many of our most intelligent representatives, we recently had convincing proof in the action of one of the Committees, to whom the bill was referred in the Legislature of Indiana. After a brief consideration, it was reportrd on adversely, and was only saved to us, at the instance of a friend of the bill, by a motion to lie upon the table. The Chairman of the Committee, a very courteous gentleman, and one of the ablest jurists of the State, having been afterwards advised that a number of the representative Dentists of the State, desired to say something in its defence, very promptly asked of the Senate the privilege of having the bill re-committed for the purpose of a hearing, which was granted. By appointment, a number of the leading practitioners from different parts of the State, appeared before the Committee, and after a patient and respectful hearing, we received, at the close of the conference, the gratifying assurance, that not only every member of the Committee, but the President of the Senate, who was present by invitation, would not only cordially vote for the measure, but advocate its passage in the Senate.

We have, unwarrantably perhaps, introduced this expose of our domestic affairs across the line, for the sole purpose of showing how nearly misapprehensions of the nature and character of our demands, subsequently corrected, came to losing us all chances of success, and for the farther purpose of illustrating the efficacy of direct means, or, in political parlance, of lobbying.

The chief misapprehension to be met with, and the one which in the first instance led to unfavorable action in our own case, is that of confounding the practice of Dentistry with general medicine, in their relation to the question of legal protection. It was claimed that inasmuch as the practice of medicine was as much exposed to the evils of quackery as that of Dentistry, and as the former was not protected or regulated by law, that *therefore*, it would be unjust and inexpedient that prohibitory enactments should be granted in favor of the latter. Now, in the first place, this objection is neither fair nor just to us since the denial of the right of protection in respect to Dentistry is predicated on the assumption, that such legislation has been *rightfully* withheld from the medical fraternity—an assumption that may be fairly challenged. Hence the *therefore* in the proposition does not either fairly or logically follow.

But we put our answer to the objection on other and more tenable grounds. Prohibitory legislation, as affecting the practice of medicine, has always, if we mistake not, been asked for in the interests of some particular school or system of medicine; and it is precisely here that such enterprises have shipwrecked, and it may be thought by many properly enough. Wide differences of opinion, both in theory and practice, obtain in all these systems. Many of them are, in this respect, radically diverse and irreconcilable. Each has its adherents and patrons among the masses, and a *monopoly* of any one in privileges must always, in the very nature of things, be regarded as a clear invasion of the inherent and natural right of the people to choose between them. Dentistry, on the contrary, presents an entirely different aspect. In its theory and practice, there is almost absolute unity and homogeneousness. There are no rival schools or systems based on differences of opinion either in theory or modes of practice. Teeth are neither inserted, filled or extracted homeopathically, allopathically or hydropathically; nor is there, we believe, distinctive modes of treating diseased conditions of these organs and their associated parts, as respects *systems* of medication. Prohibitory legislation, affecting Dental practice cannot, therefore, in any sense, be construed as favoring a monopoly. Under the terms of the bills presented, the question narrows itself down to one of simple competency or qualification, and the passage of such a law could not be regarded as *class* legislation, except as against quacks, whom it is especially designed to legally disqualify.

There is another obvious distinction between medical and Dental practice as they affect the interests of the people. Almost every neighborhood and village throughout the country has its physician, and his competency or incompetency is, in a great measure, determinable by the general success which attends his ministrations. The means employed by him are ordinarily exposed to the tests of common experience and popular familiarity with generally approved modes of medication in prevailing forms of disease, at least, and this popular and conservative intelligence must always, in some measure, act as a check to any flagrant abuse of the patient by quacks.

Another circumstance affording security to the patient, lies in the fact that, in all cases demanding medical aid, the supposed or actual failure to effect a cure or afford relief by the attending physician, may be redeemed by the advice and co-operation of counsel. The chances of securing the best results are not only thus improved, but threatened dangers to the patient by an incompetent who may have succeeded in imposing upon his confidence, may thus be averted by the timely interposition of some one more skilled. Nor does the interests of the profession itself suffer materially by the services of unauthorized persons; for in the matter of life and death, the failure of a medical man affects only his own individual reputation for skill—the faith of the people in the virtue and efficacy of medication remains fixed and unchanged.

Now, how is it in Dental practice. Resident Dentists are usually found congregated in more populous places, and patients must frequently go beyond the limits of their familiar acquaintanceship to seek among strangers the services they need. These services do not relate distinctly to the issues of life, and laboring under the belief, as many persons well informed in other matters do, that the scope and capabilities of Dentistry are comprehended chiefly in the operation of "pulling" teeth and inserting artificial ones, they are infinitely less concerned than in the choice of a physician, and fall a ready prey to the arts and seductions of quacks who lie in wait for them. With the numerical preponderance of this class, found in more inconsiderable places, and not unfrequently, perhaps, in others that aspire to metropolitan distinction, the chances are that these patients will be "taken in and done for" by some scurvy fellow whose chief distinction and highest mission it is to systematically mar and deface God's image. Let us for a moment contemplate the treatment of one of these unfortunates, taking it as a type of the usage experienced at the hands of this class.

To the practiced judgment of a skilled and educated Dentist, the case is one clearly amenable to proper treatment. One, two or three, or a half-dozen or more teeth are carious; there is some discomfort from sensitive dentine; exposure of a nerve, perhaps; tooth-ache; abscess, if you please. Ignorant of the capabilities of his art, or deliberately prostituting his acquirements, whatever they may be, to the ignoble purposes of larger profits, the unscrupulous fellow unhesitatingly condemns all the affected teeth, and unblushingly advises their indiscriminate extraction. Now, how utterly powerless and defenceless is such a patient in the hands of a man like this? Wholly uninformed of the means of relief within his reach he uncomplainingly submits to this merciless and unjustifiable act, and in all probability goes down to his grave hopelessly mutilated and unconscious of the foul wrong which has been done him. All evidences that would lead to conviction of guilt are forever veiled from the knowledge of mortals, and the consciousness of outrage, if any exists, lies buried in the breast of him who perpetrated it; and not until the last trump shall summon all men to final judgment will *accountability* be fastened upon the guilty. All evidences have perished with the sacrifice of these teeth, for who shall ever afterwards make good the accusation of malpractice against the wrong-doer? He has taken refuge in the murderous maxim, "dead men tell no tales," and there is neither remedy nor reparation. Unlike the sick room, no cry of "save me!" comes up from the patient in the hour of mortal terror, as life trembles in the balance. No reminder of accountability, conveyed in the sorrowing appeals of afflicted relatives, stays for a moment the sacrilegious hand, or awakens the conscience of the destroyer. No friendly suggestions or expressions of solicitude, volunteered by anxious friends and neighbors, warn him of the perils of failure in his duty; but like Macbeth, stealing with bloody intent into the chamber of the unconscious and confiding king, he lifts unseen his treacherous hand to execute violence upon a fellow being confiding in his truth and manhood, and in the privacy and solitude of his office, there is no eye to witness the criminal sacrifice of priceless organs, or voice to cry out against "the deep damnation of their taking off."

We have said that, as respects the interests of the profession, a physician's failure in his duty to patients affects only his individual reputation for skill. The profession of which he is a recognized member, suffers but little, if any, from his defection, for the faith of the people in medication remains comparatively unchanged, and they will be found industriously casting about for better services whenever their confidence has been betrayed or abused. Now, next to the overshadowing criminality of condemning and sacrificing teeth that might readily be redeemed and made serviceable for life, is the odium and mischief entailed upon the profession by habitually worthless and unskillful operations upon the teeth, performed ostensibly for their preservation. A physician may grossly maltreat a patient, and yet that very patient's faith in the efficacy of medication remain unshaken and unimpaired, but let him once be victimized in the matter of having his teeth filled, and the chances are that he will infect a whole neighborhood with the belief that there is no saving virtue in the operation whatever. The more uninformed portion of the people have a sort of *leveling* mode of estimating the acquirements of Dentists, and the capabilities of one are taken as a fair measure of the capabilities of the whole, and from these premises they logically enough infer that the failure of one carries with it the presumption of incapacity on the part of all. Thus any shabby fellow, with just brains enough to eat when he is hungry, may, and in countless instances does, bring into disrepute one of the most important and valuable operations in Dentistry, and every miserable abortion palmed off on the community by quacks is reflected back upon the profession to the prejudice of legitimate practice, and the humiliation, discouragement and disgust of every man, conscious of the capabilities of his art, or jealous of the respect and consideration due to himself and his calling.

We might multiply these proofs of a want of parallelism between medical and Dental practice, as they relate to the question of legal protection. What we have already said has been with no purpose of disparaging the claims of the medical profession to that legal protection from the evils of irresponsible practice which we claim for our own. While we have always thought that the best interests of the people might be advanced by wholesome legal restrictions upon the practice of medicine, we have only aimed this evening to demonstrate the yet higher claims of Dental practice to prohibitory enactments.

Speaking for and in behalf of the interests of the people, it might, at first blush, seem somewhat absurd and anomalous that legislative action should be asked which would seem to restrict them in the apparent natural right of exercising their own judgment in the choice of a Dentist, or, metaphorically speaking, of withholding from them the luxury of being burned if they are foolish enough to thrust their hands into the fire. If the masses were qualified to discriminate intelligently between the trustworthy and incompetent, we should uncomplainingly indulge them in the largest liberty of choice; but it is precisely because they are unqualified, as a general thing, to discriminate intelligently, that we advocate the passage of a law which shall stand as a wall of defense between them and imposition. That incompetents are countenanced and patronized because of the ignorance and misapprehension of the people in regard to their qualifications, is susceptible of absolute demonstration. Thus, multitudes employ quacks, but the history of human follies has never yet revealed the fact of any one having employed a quack *because* he was a quack. The employment of such an one, therefore, carries with it the irresistible and self-evident presumption of imposition and fraud; and it is in recognition, not only of the right, but of the obligation to protect the people against the self-infliction of wrong, that law-makers have repeatedly legislated in matters affecting the purely private concerns of individuals. Lotteries, for example, are declared illegal in many of the States, and thousands of persons, trusting to the false representations of these swindling concerns, who might determine, in the fancied pursuit of their private interests, to invest in such schemes, are humanely restrained in obedience to a higher intelligence which interposes and condemns such enterprizes as fraudulent. If the interests of society demand the interposition of the law to protect its members from fraud in the mere matter of dollars and cents, how much higher the duty and obligation to shield them from consequences affecting their well being for all time. We might multiply examples of this kind, but our limits forbid.

One thought more in conclusion on this subject. Viewing this movement in its bearings on the future interests of the profession, we cannot give it a too profound or thoughtful consideration. We fully recognize associated effort, expressed in general conventions and local societies, as eminently conducive to a better understanding of the principles and practice of Dentistry, and appreciate its influence in developing and enlarging the boundaries of the art and science. All men cordially unite in bearing testimony to the invaluable aids which our schools of Dentistry are yearly contributing to the ranks of the profession. Dental periodicals, as instruments of incalculable good, can hardly be over estimated. But, to our apprehension, all these agencies combined would not outweigh, in future benefits to the profession, the fruits of such prohibitory laws as are contemplated. That they would prove powerfully auxiliary to, and infuse the very essence of life into these several instrumentalities, there can be no reasonable question. Multitudes who are now hanging upon the skirts of the profession would be compelled either to abandon the practice altogether, or to avail themselves of a regular course of instruction under competent direction with a view to an examination, or betake themselves to the schools for the requisite degree. Once under such influences, there is a rational assurance that they would ultimately become worthy and reputable practitioners, an honor to their calling and respected among their fellows. Gradually the capabilities and resources of legitimate Dentistry would be known and acknowledged among all classes, and our calling would soon command everywhere that distinction among the learned, liberal and humane professions which its advanced condition and glorious achievements entitle it to. Let us hope that a consummation so devoutly to be wished is near at hand.

But we have already detained you too long, perhaps, with the discussion of a subject which may be deemed somewhat foreign to the occasion which has brought us together to-night, and will pass to some thoughts of more personal concern to yourselves.

Having completed your preparatory studies, and received from the faculty of this institution an indorsement of your fitness for the duties of the profession, you will soon disperse to your several fields of labor to assume the responsibilities of a calling in all respects worthy of your best affections and highest capacities. We speak by authority of the faculty, and of the profession at large, when we extend to you the assurance that you will carry with you, wherever you may go, their earnest and prayerful wishes for your future prosperity and success in life. The mutual regrets, natural to the breaking up of agreeable relations and associations, will at least be tempered by the consciousness of reciprocal fidelity in the discharge of duties and obligations incident alike to teacher and pupil. Having occupied for a time similar relations to former classes, we can appreciate the solicitude with which the faculty of to-day will follow each one of you into the uncertain and untried future, the records of which will, in the fullness of time, testify either for or against you, and to the honor or dishonor of the institution that has accredited you. We have an unfaltering confidence that you will remember with affection and gratitude your beloved Alma Mater, and that in return for the great benefits received at her hands, your professional lives shall honor her indorsement before all men, and enable her to proclaim with honest pride, "these are my jewels!"

The profession which you have adopted is commanding more and more the respect and confidence of community, but you should ever bear in mind that whatever degree of usefulness or consideration is accorded, it has been the fruit of personal devotion and unwearied labor generously contributed by those who have gone before you. Whatever there is of Dentistry has been handed down to you as a goodly heritage, to be received by you in trust. For such munificence you owe a debt of gratitude, and it should be your fixed and determined purpose to cancel the obligation to the uttermost extent of your abilities and opportunities, and to let your devotion to the honor and interests of your calling be such as shall, in turn, command the grateful acknowledgments of those who shall follow you.

It would be a grave and fatal mistake to go out into the world believing that you had attained to the full accomplishment of all needed acquirements within these walls. The parchments which you hold in your hands testify only to the necessary qualifications for the *commencement* of your professional career. All beyond that is a blank page on which is to be written success or failure. There is no royal road to distinction and usefulness in the republic of science and art, but every man becomes the architect of his own fortune, and must carve his way to respectability and eminence in his profession by such inflexible purposes of self-advancement as shall merit and command success.

As you journey on, every year will add new duties, responsibilities and obligations; for each succeeding year the capabilities of the Art and Science of Dentistry will be gradually unfolding, and the fields of experiment will widen and extend into unexplored regions of investigation, and these will demand continually more enlarged perceptions of the truths which underlie theories and principles, and more comprehensive aptitudes for those exigencies of practice which are always multiplied by progressive developments in all departments of science and the mechanic arts.

We are not of those who affect to believe that Dentistry, either in the extent of its acquirements or in the beneficence of its ministrations, is the common center around which revolve all other departments of the healing art, but we have a well-grounded conviction that, in the development and maturity of its resources, in the amplitude of its capabilities for good, and in the great diversity of its requirements, it is in advance of many and second to none of the specialties of medical science. If, as is generally conceded, great adaptability of capacities, and a life-time of earnest and studious application are barely sufficient to familiarize one with the complex truths and principles which enter into the practice, and to become expert in the diverse and difficult manipulations at the chair and in the laboratory, there is certainly very little hope of creditable distinction for him whom nature has disqualified, or who cheats himself into the belief that his vocation is a sinecure.

The man who, to-day, would keep abreast with his fellows in that honorable and praiseworthy competition and rivalry which contemplates the largest acquirements and the greatest measure of usefulness, must be industrious in the use of the means within his reach, and subordinate every other purpose in life to the great and central idea of completeness and thoroughness in a knowledge of all that relates to the facts and truths of his profession, and proficiency in the details of its mechanical manipulations. We would not be understood as inculcating the idea that a faithful compliance with the highest requirements of the profession necessarily implies the sacrifice of every other object or interest in life—far from it. We do not think the highest individual achievements in any profession are attained by that abnegation of self which shuts out all sources of employment or diversion not strictly tributary to the great business of life. Though we admit the force and justice of the maxim that "it is better to wear than to rust out," yet, in submitting ourselves to the former process, we prefer to do so in obedience to the prescribed laws of our being. In the intellectual as in the material world, there are ordained periods of relaxation and repose from labor, and he best subserves the divine purpose, and fulfills most perfectly the requirements of his nature who orders his life in just obedience to this law. Happily, the nature of Dental practice admits of adequate opportunities for agreeable and profitable relaxation from its more imperious duties, the sources of which are manifold. There are social interests to be cultivated. General literature; history, ancient, modern and contemporaneous have their claims. The current news of the day, as a reflex of the living present, are of personal concern to all. The study of political science, so far as it relates to the intelligent discharge of the duties of citizenship, is of binding obligation upon every man who values the blessings of free institutions. Standard works of fiction, the fine arts, music, poetry, the drama, if judiciously and rationally indulged in, may be made subservient to human happiness and usefulness, and will promote the individual in those accomplishments which adorn, and are passports to cultivated and polite society. But these objects should be held in strict subordination by him who has consecrated himself to the pursuit of science, general or special. In our own department, there are inexhaustible themes of study—themes of the highest import to us—anatomy, physiology, pathology, chemistry, Dental medicine and surgery, therapeutics, hygiene, mechanics and natural philosophy. What illimitable fields for exploration! How brief the time and opportunities for the fulfillment of duty and the achievement of the greatest good in all these broad domains of investigation and thought! You will have ample time for self-cultivation. Your first years of professional life will doubtless not be without discouragement from

"hope deferred." A full and lucrative practice is of slow growth, and it would be a personal misfortune to you, perhaps, were it otherwise, for such success at first would not only tend to enervate and demoralize by fostering a spirit of selfishness and cupidity, but would exhaust and absorb those energies of body and mind which should contribute as well to professional advancement as to pecuniary interests. If communities should prove tardy in bestowing their confidence and patronage at first, do not commit the folly of frittering away your time in worse than useless regrets, but accept your leisure hours gratefully as so many golden opportunities vouchsafed you for developing and maturing your powers, for higher and better efforts in the walks of your profession. With diligent attention to business, incorruptible integrity in all the varied relations of life, a faithful employment of all the means of self-improvement within your reach, and an inflexible purpose to excel in all the duties of your calling, your reward, not only in material profits, but in the confidence, esteem and gratitude of your fellow-men, will surely follow.

In taking leave of you, gentlemen, let us again express the earnest wish that in that future whose mysteries are, for beneficient purposes, veiled from mortal ken, you may severally realize the full and perfect fruition of all those hopes, desires and aspirations which an honorable and rational ambition begets in the breast of every man seeking for the highest good, and the applause and approbation of the wise and judicious among men.

